# Effects of Sacroiliac Joint Manipulation on Autonomic Nervous System and Lower Abdominal Pain in Women with Primary Dysmenorrhoea: A Randomized Controlled Trial

**DOI:** 10.3390/medicina60122068

**Published:** 2024-12-16

**Authors:** Sungeon Park, Hyunjoong Kim, Jihye Jung, Seungwon Lee

**Affiliations:** 1 Department of Physical Therapy, Graduate School, Sahmyook University, 815, Hwarang-ro, Seoul 01795, Republic of Korea; spring1056@naver.com; 2 Department of Senior Exercise Prescription, Gwangju Health University, Bungmun-daero 419 beon-gil, Gwangju 62287, Republic of Korea; hjkim@ghu.ac.kr; 3 Institute of SMART Rehabilitation, Sahmyook University, 815, Hwarang-ro, Seoul 01795, Republic of Korea; jihye3752@gmail.com; 4 Department of Physical Therapy, Sahmyook University, 815, Hwarang-ro, Seoul 01795, Republic of Korea

**Keywords:** dysmenorrhea, autonomic nervous system, sacroiliac joint, manipulation

## Abstract

*Background and Objectives*: Autonomic nervous system (ANS) disorders are responsible for primary dysmenorrhea and are closely linked to the spine. This study aims to evaluate the effects of sacroiliac joint manipulation on the ANS and lower abdominal pain in women with primary dysmenorrhea and proposes an alternative treatment method. *Materials and Methods*: A total of 40 participants were randomly assigned; however, 35 participants remained in the final analysis after 5 dropped out; they were assigned to either the sacroiliac joint manipulation group (*n* = 18) or the superficial heat therapy group (*n* = 17). Assessments included heart rate variability (HRV), visual analogue scale (VAS) scores for lower abdominal pain, the pressure pain threshold (PPT) of the sacroiliac joint, and the Moos Menstrual Distress Questionnaire (MMDQ) at baseline and after 4 weeks. Only the VAS and MMDQ scores were reassessed after 8 weeks to evaluate the sustained effects of the treatment. *Results*: Changes in HRV showed improvements in ANS balance only in the sacroiliac joint manipulation group (*p* < 0.05). It was found to be more effective than superficial heat therapy (*p* < 0.05). A significant decrease in lower abdominal pain following the intervention was observed in both groups (*p* < 0.05), along with the sustained effects of the treatment (*p* < 0.05). The MMDQ scores decreased after sacroiliac joint manipulation (*p* < 0.05), with greater improvements compared to the superficial heat therapy (*p* < 0.05). *Conclusions*: Sacroiliac joint manipulation positively affects ANS balance and is effective in alleviating lower abdominal pain and menstruation-related symptoms, with sustained effects observed over time. Therefore, sacroiliac joint manipulation can be an effective alternative treatment for women with primary dysmenorrhea.

## 1. Introduction

Primary dysmenorrhea, in the absence of pelvic pathology, is defined as painful cramps in the lower abdomen that occur just before or during menstruation in most women [1,2]. It is associated with absenteeism from school or work, the disruption of one’s daily life, and high social costs [3,4]. Primary dysmenorrhea occurs due to the excessive secretion of prostaglandin by endometrial cells during menstruation. The primary role of prostaglandins is to induce uterine muscle contractions. Excessive contractions of uterine muscle lead to ischemia, resulting in insufficient oxygen supply to the uterine tissues. Consequently, endometrial cells die and blood vessels within the endometrium are damaged, causing pain and cramping [4,5,6]. Particularly, when the uterine contraction pressure exceeds the arterial blood pressure, ischemia intensifies, leading to the production of anaerobic metabolic by-products that stimulate C-type nociceptive nerves, thereby exacerbating pain [7]. However, the physiopathology of dysmenorrhea is not yet fully understood [8].

Autonomic nervous system (ANS) imbalance caused by sympathetic overactivity could be another cause of primary dysmenorrhea [9]. In response to changes in the external environment, the ANS maintains the homeostasis of the internal environment of the human body through the interaction between sympathetic and parasympathetic nerves. A lack of harmony between the two systems may cause various symptoms [10]. A previous study reported that healthy women and women with dysmenorrhea show different responses to the ANS [11]. A similar study found that women with dysmenorrhea showed a significant increase in sympathetic nerve activity and a significant decrease in parasympathetic nerve activity during the late luteal phase, as compared to those without dysmenorrhea [10,12,13]. The above findings suggest that women with severe dysmenorrhea may have a dysregulated ANS [9]. The sympathetic and parasympathetic nerve pathways are closely associated with the spinal vertebrae, in particular, with the second to fourth sacral segments (S2–S4) and the tenth thoracic (T10) to the first lumbar segments (L1) [14]. Therefore, the malalignment of the spine is associated with ANS dysfunction [15], and restoring spinal alignment in the sagittal plane is considered to play an important role in improving the functioning of the ANS [16].

The anterior and posterior inclinations of the sacrum may influence not only the fifth lumbar vertebra but also the upper spinal segments [17], of which the tenth thoracic to first lumbar vertebrae and second to fourth sacral vertebrae are closely connected to the sympathetic and parasympathetic nerves [14]. Additionally, the sacrum is neurologically and mechanically connected to the uterus through the pelvic splanchnic nerve and uterosacral ligament [8,18,19]. Although the anatomical structure of the sacroiliac joint may vary among individuals [20], differences may exist in ANS responses. However, it is an important area where potential effects on all spinal joint functions, autonomic nervous system balance, and biomechanical movement can be expected [21,22].

A previous study reported that lower abdominal pain associated with dysmenorrhea may be correlated with sacral slope and pelvic incidence in women with primary dysmenorrhea [23]. Another study found that the manipulation of the sacroiliac joint in women with primary dysmenorrhea improved ANS balance and reduced lower back pain [24].

Nonsteroidal anti-inflammatory drugs (NSAIDs) and hormonal medications such as contraceptives are commonly used as initial treatments for dysmenorrhea [25]; however, the treatment failure rate may still be as high as 20–25% [26], leading many women to seek alternatives to traditional medical treatments [8]. Exercise therapy, which is a low-cost and side-effect-free alternative for reducing dysmenorrhea symptoms, is being utilized [27]; however, in some patients, symptoms may worsen or abnormal responses may occur after exercise [28]. Another alternative treatment for primary dysmenorrhea is spinal manipulation, which is suggested to have fewer side effects compared to pharmacological treatments [29]. Spinal manipulation influences the ANS [30], and a systematic review of spinal manipulation therapy applied to women with dysmenorrhea found that this therapy may be effective in relieving pain [31].

Spinal manipulation to influence the ANS seems to be a good alternative treatment for women with primary dysmenorrhea. However, previous studies that demonstrated the effect of the manipulation on dysmenorrhea performed general spinal manipulations rather than interventions in a specific spinal segment. Only a few studies to date have identified changes in the ANS following interventions. Therefore, this study aimed to evaluate the effects of sacroiliac joint manipulation on the autonomic nervous system through heart rate variability and examine its impact on lower abdominal pain in women with primary dysmenorrhea.

## 2. Materials and Methods

### 2.1. Participant Description

This study was conducted on women living in Seoul who suffer from dysmenorrhea. Because women aged ≤ 30 years are most commonly affected by primary dysmenorrhea, the participants selected for this study were aged 20–29 years [14]. Selection criteria for the participants included those with a regular menstrual cycle (24–32 days) who have had primary dysmenorrhea for at least 1 year, dysmenorrhea-related lower abdominal pain, a visual analysis scale (VAS) score ≥ 5, a body mass index of 20–30, and a positive Gillet test for the sacroiliac joint [32,33]. Exclusion criteria included women with gynaecological conditions such as pelvic inflammatory disease, uterine leiomyoma, polycystic ovarian syndrome, and endometriosis; those with an intrauterine contraceptive device; and those taking contraceptives or non-steroidal anti-inflammatory drugs at the time of the experiment. Additionally, those who had had spinal manipulation performed on them within 1 month of the experiment, as well as those who had contraindications to spinal manipulation or experienced fear or stress related to it, were also excluded [24].

Based on previous studies that manipulated the pelvis of women with primary dysmenorrhea, Cohen’s f value of 0.285 was obtained [33]. The power was set to 0.80 with a significance level of 0.05. Using the G*power 3.1.9.6 (Franz Faul, Universität Kiel, Germany) program, the final required sample size was calculated to be 28 participants. However, to minimize the impact of variables and uncertainties, such as the menstrual cycle, 40 participants were recruited, considering a 30% dropout rate. The appropriate sample size was determined to ensure the statistical power and reliability of the study.

### 2.2. Procedure

The participants were recruited through leaflet advertisements from T clinic, T and M Pilates Center, located in Seoul, between 6 March 2023 and 29 March 2023. The trial commenced on 30 April 2023, after the recruitment was completed. All participants received a detailed explanation of the purpose and details of the study, and their voluntary consent was obtained through signing a relevant consent form. The study protocol was approved by the institutional review board of Sahmyook University (SYU 2023-01-002-002). The protocol was registered at ClinicalTrials.gov (NCT05752864). The study was conducted according to the guidelines of the Declaration of Helsinki.

Because participants had generally complained of pain on the first menstrual day in previous studies [34], the evaluation period was set to the first day of menstruation ± 2 (baseline). The heart rate variability, VAS for pain in the lower abdomen, and pressure pain threshold (PPT) of the intervened sacroiliac joint were measured. In addition, the Moos Menstrual Distress Questionnaire (MMDQ) was used to measure the onset of menstruation-related symptoms and the severity level. Following a 4-week intervention administered twice weekly, participants were reassessed on the first day of menstruation of the next cycle ± 2 days (4 weeks). After the experiment, VAS and MMDQ scores were reassessed on the first day of menstruation of the following cycle ± 2 (8 weeks).

**Figure 1:**
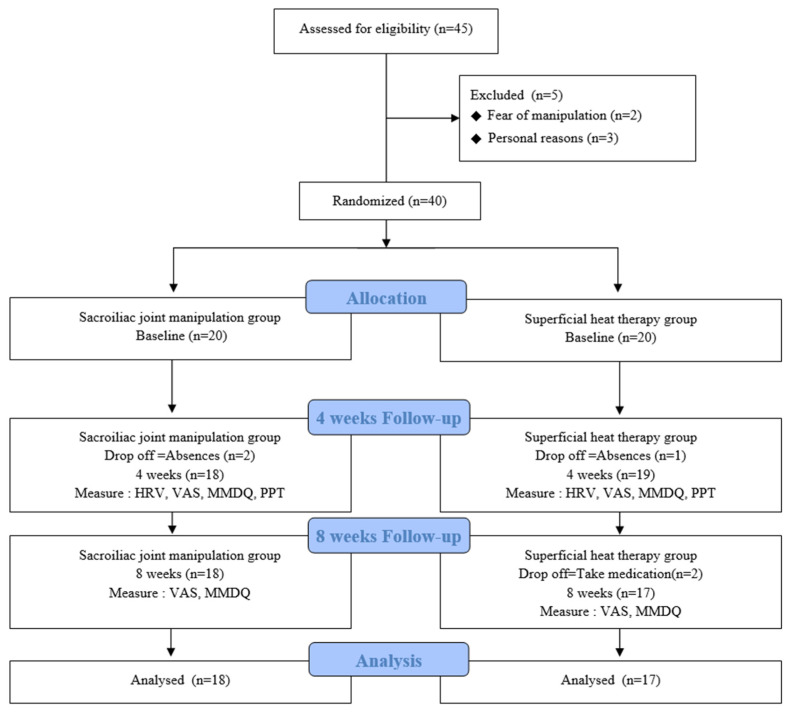
Study procedure.

A total of 45 participants were assessed for eligibility. Five participants were excluded due to the fear of manipulation (*n* = 2) and personal reasons (*n* = 3), resulting in 40 eligible participants. The sacroiliac joint manipulation group (*n* = 20) and superficial heat therapy group (*n* = 20) were created through random allocation. The random allocation sequence was entered into Microsoft Excel 2016 (Microsoft; Redmond, WA, USA) and included randomly permuted blocks of 4 with an allocation ratio of 1:1. Two participants dropped out from the sacroiliac joint manipulation group due to absence, whereas three people dropped out from the superficial heat therapy group due to absence (*n* = 1) and taking medication (*n* = 2) (Figure 1). Participant enrolment and the intervention were conducted by the principal investigator, who had at least 10 years of experience, whereas the evaluation and collection of basic results were conducted by another physical therapist, who was blinded to the group allocation.

### 2.3. Intervention

The treatment area for sacroiliac joint manipulation was determined after evaluating the function of both sacroiliac joints through the Gillet test. The Gillet test is performed while the participants stand. The therapist positions one thumb on the second sacral spinous process and the other thumb on the posterior superior iliac spine. The participants are instructed to lift their leg, causing maximum hip flexion. Usually, the posterior superior iliac spine (PSIS) moves inferiorly relative to the second sacral spinous process. The PSIS was considered positive if it did not move caudally toward the sacrum [35]. The process was subsequently repeated with the opposite leg. The principal investigator with 10 years of experience independently performed all interventions to minimize inter-practitioner variability. The investigator was thoroughly trained in standardized intervention protocols before conducting the research. The manipulation technique applied to the sacroiliac joint utilized the high-velocity, low-amplitude (HVLA) technique. The application of this technique typically occurs within <200 ms [36]. The participants were made to lie on their sides with the sacroiliac joint facing upward. The therapist stood in front of the participant, bent the participant’s top knee at a 90-degree angle, and positioned their top foot on the popliteal fossa of the bottom leg. The therapist fixed the participants’ top leg in front of their thighs and gently pulled the participants’ hind arm, placing it on the table. The therapist placed his one hand on the patient’s top elbow to fix it, placed the hypothenar eminence of the other hand on the PSIS, and applied an HVLA thrust in a caudal and lateral line of drive along the plane of the ilium [37].

Superficial heat therapy is a common intervention method used for dysmenorrhea [15]. Participants received a hot pack (40–45 °C) on the lower abdomen for 20 min while in the supine position [38]. Unlike other treatments for primary dysmenorrhea, no adverse side effects have been reported with manual lumbar spine therapy, except for a slight burning sensation in the lumbar region [33].

### 2.4. Outcomes

The primary outcome is heart rate variability (HRV), which serves as a non-invasive evaluation method to assess the functioning of the ANS [34]. HRV analysis emphasizes changes in heart rate patterns, providing a detailed assessment of the autonomic regulation of the heart [39]. It was measured in a manner similar to the HRV measurement protocol used in previous studies [40]. HRV is measured using an autonomic nervous balance tester (SA3000 new, Medicore Co, Gyeonggi-do, Korea). Participants had their HRV measured for 3 min in a supine position with electrodes attached to their left arm, left leg, and right leg. Based on previous studies indicating that HRV measurements of less than 2 min are reliable, the measurement was conducted for 3 min, considering the time required for the heart rate variability measurement device to accurately assess the participants’ HRV [41]. We measured the time domain standard deviation of all normal P-P intervals, square root of the mean of the sum of the squares of differences between adjacent N-N intervals, frequency domain total power, low frequency (LF), and high frequency (HF) [34]. Additionally, autonomic nervous system activity and balance were automatically recorded in the analyser through data processing based on HRV-measured values. These measurements provide valuable information; however, careful interpretation is needed as HRV is influenced by multiple interacting factors including physiological/pathological, lifestyle, environmental, and neuropsychological aspects [42]. To minimize confounding variables, measurements were conducted in a temperature-controlled room (20–25 °C). Additionally, excessive exercise, smoking, and drinking were prohibited within 12 h of measurement.

The secondary outcomes were measured using pain-related VAS, PPT, and MMDQ. The VAS can be applied to different groups experiencing pain, including women with menstrual pain [4,43]. VAS was used in this study to measure the intensity of lower abdominal pain. A 100 mm line has “no pain” indicated at one end and “worst pain ever experienced” indicated at the other end. In this study, a hand-held digital force gauge (YST-100, CNYST, Anhui, China) with a probe diameter of 2 cm was used to measure PPT at the sacroiliac joint that was shown to be positive on the Gillet test. The participants in the prone position were asked to say “now” at the exact moment they felt pain [44,45]. MMDQ is used to measure the onset of menstrual symptoms and their severity levels [46]. Based on previous studies, 37 MMDQ items for pain, such as water retention, automatic reactions, negative affectivity, impaired concentration, and behavioural changes, were implemented in this study [47]. The participants rated their menstrual discomfort from 0 (”not at all”) to 5 (“extremely severe”).

### 2.5. Data Analysis

Windows SPSS ver. 25.0 software (SPSS Inc., Chicago, IL, USA) was used for data analysis and statistical processing. To assess the normality of the collected data, a Kolmogorov–Smirnov test was conducted, and an independent *t*-test was performed to confirm homogeneity between groups. A paired *t*-test was employed to examine within-group variations in HRV and PPT after the intervention, and an independent *t*-test was used to examine inter-group differences. The significance level was set at *p* < 0.05.

Additionally, a 2 × 3 repeated-measures analysis of variance (ANOVA) was carried out on the VAS and MMDQ scores for lower abdominal pain to assess the impact of sacroiliac joint manipulation over time. When a significant within-group interaction was found over time, a paired *t*-test was used to compare the changes at baseline, 4 weeks, and 8 weeks. If a significant interaction between time and group was found, an independent *t*-test was performed to determine the difference between the sacroiliac joint manipulation and superficial heat therapy groups at the time points of 4 weeks and 8 weeks. The significance level was set at 0.017 using the Bonferroni correction. The effect size (Cohen’s d) and 95% confidence interval (CI) for variables were obtained. To address missing data, additional analyses were conducted focusing solely on VAS, MMDQ, and ANS balance scores. The baseline observation carried forward method was applied, and the analysis was conducted using the intention-to-treat (ITT) approach.

## 3. Results

The final 35 participants (sacroiliac joint manipulation group: *n* = 18; superficial heat therapy group: *n* = 17) were analysed in this study. The general characteristics of the sacroiliac joint manipulation and superficial heat therapy groups were similar. Table 1 presents the general characteristics of the participants.

### 3.1. Changes in HRV and PPT of Sacroiliac Joint Based on Intervention

In the within-group change in HRV from the baseline to 4 weeks, ANS balance decreased significantly only in the sacroiliac joint manipulation group (d = 0.97; 95% confidence interval (CI): 13.92, 67.30). A significant between-group difference was observed for ANS balance in the sacroiliac joint manipulation group (d = 1.11; 95% CI: 20.66, 87.15) (Table 2).

### 3.2. Change in PPT of Sacroiliac Joint Based on Intervention

The PPT of the sacroiliac joint indicated no significant within-group or inter-group differences at baseline to 4 weeks (*p* > 0.05) (Table 2).

### 3.3. Changes in Lower Abdominal Pain and MMDQ Based on Intervention

**Figure 2:**
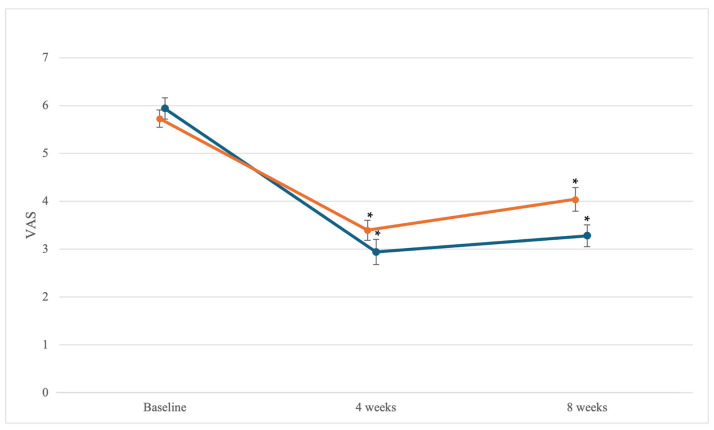
VAS changes in the sacroiliac joint manipulation (blue line) and superficial heat therapy groups (orange line) over time. Error bars represent standard error. Both groups showed significant differences over time (*p* < 0.05). Based on the results of checking the within-group differences, both groups showed significant differences from baseline to 4 weeks and from baseline to 8 weeks (* *p* < 0.017). No differences were observed between the groups over time. * Indicates statistical significance at *p* < 0.017 for the within-group comparison with Bonferroni’s correction.

Table 3 shows the VAS and MMDQ scores for lower abdominal pain in the sacroiliac joint manipulation and superficial heat therapy groups. The VAS scores showed within-group differences over time in both groups (sacroiliac joint manipulation, *F* = 41.88; *p* = 0.001; superficial heat therapy, *F* = 33.64; *p* = 0.001). A paired *t*-test was used to determine within-group differences over time, and significant differences were observed from the baseline to 4 weeks and from the baseline to 8 weeks between the two groups. In the sacroiliac joint manipulation group, significant differences were observed between the scores at baseline and at 4 weeks (d = 2.92; 95% CI: 2.43, 3.56) and between the scores at baseline and at 8 weeks (d = 2.81; 95% CI: 1.97, 3.37). In the superficial heat therapy group, significant differences were also found between the scores at baseline and at 4 weeks (d = 2.89; 95% CI: 1.91, 2.80) and between the scores at baseline and at 8 weeks (d = 1.89; 95% CI: 1.08, 2.33). No significant relation was observed between the time and pain levels of the groups (*F* = 2.57; *p* = 0.088) (Figure 2).

**Figure 3:**
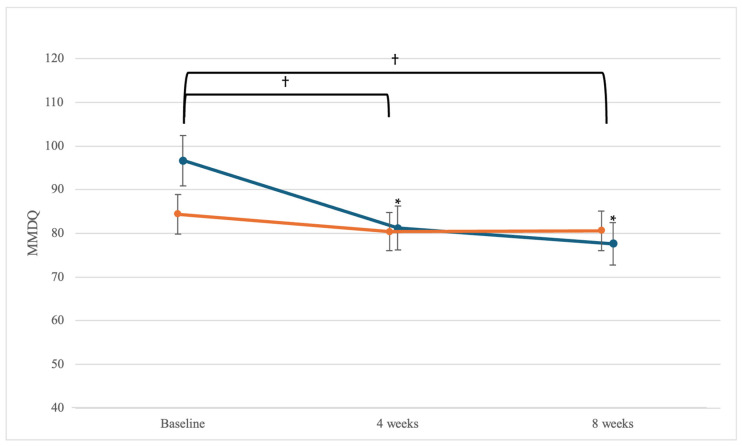
MMDQ changes in sacroiliac joint manipulation (blue line) and superficial heat therapy groups (orange line) over time. Error bars represent standard deviation. The sacroiliac joint manipulation group showed significant differences over time from baseline to 4 weeks and from baseline to 8 weeks (* *p* < 0.017). Significant differences were observed between the groups over time at 4 weeks and at 8 weeks (^†^ *p* < 0.05). * Indicates statistical significance at *p* < 0.017 for within-group comparison with Bonferroni’s correction. † Indicates statistical significance at *p* < 0.05 for between-groups comparison.

The MMDQ scores showed significant within-group differences over time in the sacroiliac joint manipulation group (*F* = 15.63; *p* = 0.001). A paired *t*-test was used to check the within-group differences over time, and significant differences were present from the baseline to 4 weeks (d = 0.67; 95% CI: 7.35, 23.54) and from the baseline to 8 weeks (d = 0.84; 95% CI: 10.91, 27.19). A significant interaction was also observed between the time and pain levels of the group (*F* = 7.49; *p* = 0.001). An independent *t*-test was used to check within-group differences over time, and there were significant differences between 4 weeks and 8 weeks (4 weeks: *d* = 0.91; 95% CI: 2.66, 20.23; 8 weeks: *d* = 1.23; 95% CI: 6.61, 23.86) (Figure 3). This means that the sacroiliac joint manipulation reduced the dysmenorrhea symptoms of participants and proved to be more effective compared to the superficial heat therapy.

### 3.4. Evaluation of VAS, MMDQ, and ANS Balance via ITT Analysis

Table 4 shows the HRV, VAS, and MMDQ scores for lower abdominal pain in the sacroiliac joint manipulation and superficial heat therapy groups, analysed using the ITT. 

For the within-group change in HRV, a significant decrease in ANS balance was observed only in the sacroiliac joint manipulation group (d = 0.83; 95% CI: 12.08, 61.02). A significant difference between the groups was observed (*p* = 0.002; 95% CI: 18.16, 77.54).

The VAS scores revealed significant within-group variations over time (sacroiliac joint manipulation, *F* = 41.89 and *p* = 0.001; superficial heat therapy, *F* = 33.64 and *p* = 0.001). A paired *t*-test was used to determine within-group differences over time, and significant differences were observed from the baseline to 4 weeks and from the baseline to 8 weeks between the two groups. In the sacroiliac joint manipulation group, significant differences were observed between the scores at baseline and at 4 weeks (d = 2.02; 95% CI: 2.04, 3.37) and between the scores at baseline and at 8 weeks (d = 1.95; 95% CI: 1.67, 3.14). In the superficial heat therapy group, significant differences were also found between the scores at baseline and at 4 weeks (d = 1.83; 95% CI: 1.45, 2.55) and between the scores at baseline and at 8 weeks (d = 1.39; 95% CI: 10.85, 2.05). No significant relation was observed between the time and pain levels of the groups (*F* = 2.59; *p* = 0.082).

The MMDQ scores presented significant within-group differences over time in the sacroiliac joint manipulation group (*F* = 14.40; *p* = 0.001). A paired *t*-test was used to check the within-group differences over time, and significant differences were present from the baseline to 4 weeks (d = 0.60; 95% CI: 6.36, 21.44) and from the baseline to 8 weeks (d = 0.74; 95% CI: 9.40, 24.44). A significant interaction was also observed between the time and pain levels of the group (*F* = 7.62; *p* = 0.001). An independent *t*-test was used to check the within-group differences over time, and there were significant differences between the scores at 4 weeks and at 8 weeks (4 weeks: 95% CI: 2.56, 18.44; 8 weeks: 95% CI: 5.78, 22.02). 

## 4. Discussion

This study investigated the effects of a manipulation applied to the sacroiliac joint on the ANS and lower abdominal pain in women with primary dysmenorrhea.

### 4.1. Effect of Sacroiliac Joint Manipulation on the ANS

A significant change in ANS balance was observed in the sacroiliac joint manipulation group as compared to the superficial heat therapy group. ANS balance is expressed as a quantifiable value, measured as the ratio of LF to HF; the value ranges from 0 to 150, in which values ≤50 are included in the normal category [44].

Systemic reviews of previous studies have analysed the results and demonstrated that spinal manipulation has a significant influence on the ANS [48,49] and that the response of the ANS varies depending on the spinal region being manipulated [50]. Even when spinal manipulations are applied to the same region, different studies have reported varying results. For example, Bergel and Paulus [51] reported a dominant sympathetic response, whereas Zhang et al. [52] reported parasympathetic dominance. Overall, the impact of spinal manipulation on the ANS has been established [53]. Alterations in somatosensory processing following spinal manipulation have been reported to occur, particularly within the prefrontal cortex [54]. The prefrontal cortex is connected to supraspinal structures related to autonomic function, including the cerebellar vermis, anterior cingulate cortex, and insular cortex [55,56], and spinal manipulation may influence autonomic function through these structures [54]. A cross-sectional study in women with primary dysmenorrhea investigating changes in HRV with sacroiliac joint manipulation found significant changes in the ANS balance within a short period, which is consistent with our findings [24].

The freedom of sacral movement may contribute to maintaining ANS balance by influencing the parasympathetic activity of the second to fourth sacral ventral rami [57]. In addition, spinal manipulation, when used as an intervention, induces changes in neural discharge in muscle mechanoreceptors [58], which may have important effects on motor control related to alterations in spinal alignment [59]. Therefore, in this study, the significant changes in the ANS balance observed in women with primary dysmenorrhea suggest that sacroiliac joint manipulation may have potentially affected the alignment of the pelvis and spine in women with dysmenorrhea.

### 4.2. Effect of Sacroiliac Joint Manipulation on Low Abdominal Pain

In this study, both groups indicated a decreasing trend for lower abdominal pain over time. Holtzman et al. [60] showed that lumbosacral manipulation reduced menstrual pain. Another study found that spinal manipulation in women with premenstrual syndrome significantly reduced abdominal and lower back pain in the lumbar region [61], which agreed with the results of previous reports. These results suggest that the spinal manipulation of restricted joints creates normal joint movement, inhibits excessive uterine contractions, and increases blood flow [5]. Additionally, the HVLA technique used in this study appears to contribute to pain relief by modulating descending pain regulation [62]. The method of descending pain regulation is a specific process in which ascending nociceptive signals in the spinal cord are inhibited through pathways descending from the brainstem. Originating in the periaqueductal gray matter of the midbrain, this method reduces pain sensitivity by inhibiting pain signals at the spinal cord level. Through this mechanism, the HVLA technique induces mechanical hypoalgesia, leading to a significant decrease in pain [63]. The ANS interacts with the pain receptors [64]; a few neurons in the uterosacral ligament, which mechanically connects the sacrum to the uterus, are included in the sympathetic nerve fibres that arise from T10–L1 [65]. Therefore, it is likely that the sacroiliac joint manipulation used in this study affected the sacrum, which in turn affected the uterosacral ligament. It is thought that these changes may have a positive effect on the ANS, thereby bringing relief from lower abdominal pain.

To assess the persistent therapeutic effects of VAS, a comparative analysis of scores at baseline, 4 weeks, and 8 weeks within the groups was performed, and significant differences were observed in both groups (*p* < 0.05). No significant correlation was found between time and group (*p* > 0.017). The post-test results indicated a change of 3.47 cm after 4 weeks and 3 cm after 8 weeks in the sacroiliac joint manipulation group, which met the requirement for a minimal clinically important difference (MCID) of 3 cm [66]. In contrast, the superficial heat therapy group showed a decrease of 2.5 cm after 4 weeks and 1.71 cm after 8 weeks; however, it did not meet the MCID threshold. These results suggest that sacroiliac joint manipulation has a clinically more significant effect in relieving lower abdominal pain compared to superficial heat therapy.

### 4.3. Effect of Sacroiliac Joint Manipulation on MMDQ

The severity and onset of menstruation-related symptoms were confirmed using the MMDQ, and significant within-group differences were observed over time (*p* < 0.05). Subsequently, a comparative analysis was conducted using the post-hoc test from the baseline to 4 weeks and from the baseline to 8 weeks. Significant differences were observed only in the sacroiliac joint manipulation group (*p* < 0.05) at the baseline to 4 weeks and at the baseline to 8 weeks. Significant differences were present depending on the time and group; significant differences were also observed in the second and third cycles (*p* < 0.017). This means that sacroiliac joint manipulation reduced the dysmenorrhea symptoms of participants and proved to be more effective compared to the superficial heat therapy. Gurav and Nahar’s [67] study confirmed the effect of lumbar spine manipulation on menstrual distress and showed an improvement in MMDQ scores, which is consistent with the results of this study. The MMDQ consists of eight categories: pain, concentration, behavioural changes, automatic reactions, water retention, negative affectivity, arousal, and control [46]. Positive changes in the ANS and pain relief in the lower abdomen after sacroiliac joint manipulation affected the category of pain and ANS response in the MMDQ, indicating that the score had a significant effect.

### 4.4. Effect of Sacroiliac Joint Manipulation on PPT

The PPT of the sacroiliac joint was similar in both groups. In our study, the PPT before the intervention was 81.79 N (8.34 kg/cm²) in the sacroiliac joint manipulation group and 78.66 N (8.02 kg/cm²) in the superficial heat therapy group. Based on previous studies, the PPT of the healthy participants was 83.35 N (8.5 kg/cm²), whereas that of the participants with sacroiliac joint pain was 23.53 N (2.4 kg/cm²) [68]. The participants in this study presented PPT values that were nearly identical to those of healthy individuals, which suggests that women in this study did not have any specific pain in the sacroiliac joint. This is presumed to explain the lack of significant change in the PPT of the sacroiliac joint.

This study had several limitations. First, there were limitations in controlling lifestyle factors that could affect HRV, such as smoking [69] and a reduction in physical activity [70,71]. Second, although the participants in this study presented PPT values similar to those of healthy individuals, it is important to consider the possibility that sacroiliac joint dysfunction can also contribute to lower abdominal pain [72,73]. Therefore, future studies should further investigate the distinction between sacroiliac joint dysfunction and primary dysmenorrhea to clarify the potential overlap of symptoms.

## 5. Conclusions

In women with primary dysmenorrhea, sacroiliac joint manipulation significantly alters the balance of the ANS. In addition, it reduces lower abdominal pain and alleviates dysmenorrhea symptoms. This intervention protocol was found to be effective in alleviating the pain of primary dysmenorrhea, as the pain-reducing effect persisted in the subsequent menstrual cycle. Due to the limited sample size and design limitations of this study, caution is needed in interpreting these conclusions. Further studies with larger sample sizes and diverse populations are necessary to verify the long-term effects of sacroiliac joint manipulation on primary dysmenorrhea. Overall, sacroiliac joint manipulation can be considered as one of the treatment options for primary dysmenorrhea.

## Figures and Tables

**Table 1:** General characteristics of participants.

Characteristic	Sacroiliac Joint Manipulation (*n* = 18)	Superficial Heat Therapy (*n* = 17)	t/χ²	*p*-Value
Age (year)	23.72 ± 2.19 ^a^	24.00 ± 2.50	−0.350	0.730
Height (cm)	160.17 ± 5.13	161.18 ± 5.28	−0.573	0.570
Weight (kg)	56.17 ± 5.62	56.59 ± 4.72	−0.241	0.811
BMI (kg/m^2^)	21.85 ± 1.43	21.83 ± 2.19	0.028	0.978
Menstrual cycle (days)	29.06 ± 2.31	29.88 ± 2.20	−1.081	0.287
Positive on Gillet Test (Rt/Lt)	(7/11)	(9/8)	6.369 ^b^	0.173

Abbreviations: BMI, body mass index; Lt, left; Rt, right. ^a^ Values are presented as mean ± standard deviation. ^b^ Chi-square test.

**Table 2:** Heart rate variability by intervention.

Outcome/Time Point	Sacroiliac Joint Manipulation (*n* = 18)	Superficial Heat Therapy (*n* = 17)	Mean Difference Between Groups (95% CI)	*p*-Value/Effect Size
SDNN (ms)				
Baseline	41.39 ± 13.23 ^a^	39.53 ± 11.88		0.281 *Cohen’s d* = 0.37
4 weeks	41.90 ± 17.35	35.23 ± 13.04		
Baseline to 4 weeks	−0.51 (−7.93, 6.91) ^b^	4.30 (−1.25, 9.84)	−4.80 (−13.74, 4.13)	
RMSSD (ms)				
Baseline	40.63 ± 19.19	35.04 ± 20.91		0.751 *Cohen’s d* = 0.11
4 weeks	37.44 ± 22.00	30.37 ± 17.74		
Baseline to 4 weeks	3.19 (−5.24, 11.61)	4.67 (−0.23, 9.56)	−1.48 (−10.94, 7.98)	
TP (ms^2^)				
Baseline	7.07 ± 0.70	6.79 ± 0.65		0.959 *Cohen’s d* = 0.02
4 weeks	6.79 ± 1.07	6.53 ± 0.79		
Baseline to 4 weeks	0.27 (−0.26, 0.80)	0.26 (−0.15, 0.66)	0.02 (−0.62, 0.66)	
LF (ms^2^)				
Baseline	5.67 ± 0.73	5.27 ± 0.75		0.541 *Cohen’s d* = 0.21
4 weeks	5.60 ± 0.94	4.89 ± 0.74		
Baseline to 4 weeks	0.08 (−0.53, 0.68)	0.29 (−0.12, 0.70)	−0.21 (−0.92, 0.49)	
HF (ms^2^)				
Baseline	5.67 ± 1.36	5.13 ± 1.17		0.859 *Cohen’s d* = 0.06
4 weeks	5.27 ± 1.30	4.78 ± 1.28		
Baseline to 4 weeks	0.40 (−0.08, 0.89)	0.35 (−0.05, 0.75)	0.05 (−0.55, 0.66)	
ANS Activity (score)				
Baseline	91.39 ± 11.29	86.71 ± 9.48		0.996 *Cohen’s d* = 0.00
4 weeks	88.00 ± 16.31	83.29 ± 11.95		
Baseline to 4 weeks	3.39 (−4.49, 11.27)	3.41 (−2.63, 8.99)	−0.02 (−9.35, 9.31)	
ANS Balance (score)				
Baseline	77.56 ± 46.56	59.82 ± 29.84		0.002 * Cohen’s d = 1.11
4 weeks	36.94 ± 36.77	73.12 ± 29.35		
Baseline to 4 weeks	40.61 (13.92, 67.30) *	−13.29 (−35.16, 8.57)	53.91 (20.66, 87.15)	
PPT (N)				
Baseline	81.79 ± 7.55	78.66 ± 10.07		0.981 *Cohen’s d* = 0.08
4 weeks	82.84 ± 7.24	79.69 ± 10.39		
Baseline to 4 weeks	−1.05 (−2.68, 0.58)	−1.02 (−2.60, 0.56)	−0.03 (−2.21, 2.16)	

Abbreviations: HF, high frequency; LF, low frequency; PPT, pressure pain threshold; RMSSD, square root of the mean of the sum of the square of differences between adjacent NN intervals; SDNN, standard deviation of all normal P-P intervals; TP, total power; ANS, autonomic nervous system. ^a^ Values are presented as mean ± standard deviation. ^b^ Change values are presented as difference (95% confidence interval). * Indicates statistical significance at *p* < 0.05.

**Table 3:** Changes in lower abdominal pain and MMDQ by intervention.

Outcome/Time Point	Sacroiliac Joint Manipulation (*n* = 18)	Superficial Heat Therapy (*n* = 17)	Mean Difference Between Groups (95% CI)	Time × Group F (*p*)
VAS (cm)				
Baseline	5.94 ± 0.94 ^a^	5.76 ± 0.75		2.57 (0.088)
4 weeks	2.94 ± 1.11	3.41 ± 0.87		
8 weeks	3.28 ± 0.96	4.06 ± 1.03		
Baseline to 4 weeks	3.00 (2.43, 3.56) ^b,^*	2.35 (1.91, 2.80) *	0.65 (−0.05, 1.34)	
Baseline to 8 weeks	2.67 (1.97, 3.37) *	1.71 (1.08, 2.33) *	0.96 (0.05, 1.87)	
MMDQ (score)				
Baseline	96.67 ± 24.33	84.59 ± 19.02		7.49 (0.001) ^‡^
4 weeks	81.22 ± 21.43	80.59 ± 18.27		
8 weeks	77.61 ± 20.80	80.76 ± 18.58		
Baseline to 4 weeks	15.44 (7.35, 23.54) *	4.00 (0.21, 7.80)	11.44 (2.66, 20.23) ^†^	
Baseline to 8 weeks	19.06 (10.91, 27.19) *	3.82 (0.60, 7.01)	15.23 (6.61, 23.86) ^†^	

Abbreviations: MMDQ, Moos Menstrual Distress Questionnaire; VAS, visual analogue scale. ^a^ Values are presented as mean ± standard deviation. ^b^ Change values are presented as difference (95% confidence interval). * Indicates statistical significance at *p* < 0.017 in within-group comparison with Bonferroni‘s correction. ^†^ Indicates statistical significance at *p* < 0.05 in the between-groups comparison. ^‡^ Indicates statistical significance at *p* < 0.05 for time × group interaction.

**Table 4:** Evaluation of VAS, MMDQ, and ANS balance through intention-to-treat analysis.

Outcome/Time Point	Sacroiliac Joint Manipulation (*n* = 20)	Superficial Heat Therapy (*n* = 20)	Mean Difference Between Groups (95% CI)	*p*-Value/Effect Size
ANS Balance (score)				
Baseline	81.30 ± 45.55 ^a^	66.05 ± 31.36		0.002 ^*^ Cohen’s d = 1.03
4 weeks	44.75 ± 42.30	77.35 ± 28.89		
Baseline to 4 weeks	3.13 (12.08, 61.02) ^b^*	−1.29 (−29.70, 7.10)	47.85 (18.16, 77.54)	
VAS (cm)				
Baseline	6.05 ± 0.95	5.85 ± 0.75		2.59 ^c^ (0.082)
4 weeks	3.35 ± 1.63	3.85 ± 1.35		
8 weeks	3.65 ± 1.46	4.40 ± 1.27		
Baseline to 4 weeks	8.52 (2.04, 3.37) **	7.65 (1.45, 2.55) **	0.70 (−0.13, 1.53)	
Baseline to 8 weeks	6.84 (1.67, 3.14) **	5.08 (0.85, 2.05) **	0.95 (0.03, 1.87)	
MMDQ (score)				
Baseline	98.30 ± 23.82	88.80 ± 20.37		7.62 ^‡^ (0.001)
4 weeks	84.40 ± 22.79	85.40 ± 20.85		
8 weeks	81.15 ± 22.77	85.55 ± 20.77		
Baseline to 4 weeks	3.86 (6.36, 21.44) **	2.19 (0.15, 6.65)	10.50 (2.56, 18.44) ^†^	
Baseline to weeks	4.63 (9.40, 24.44) **	2.45 (0.48, 6.03)	13.90 (5.78, 22.02) ^†^	

^a^ Values are presented as mean ± standard deviation. ^b^ Change values are presented as difference (95% confidence interval). ^c^ Values for F (p) related to the interaction between time and groups are presented. * Indicates statistical significance at *p* < 0.05. ** Indicates statistical significance at *p* < 0.017 for within-group comparison with Bonferroni‘s correction. ^†^ Indicates statistical significance at *p* < 0.017 for within-group comparison with Bonferroni’s correction. ^‡^ Indicates statistical significance at *p* < 0.05 for time × group interaction.

## Data Availability

The data presented in this study are available on request from the corresponding author.
